# Forum: Oral History in der Medizin. Etwas Besonderes?

**DOI:** 10.1007/s00048-024-00380-7

**Published:** 2024-02-27

**Authors:** Felicitas Söhner, Agnès Arp, Thorsten Halling, Nils Hansson, Uta Hinz, Nils Löffelbein, Constanze Schliwa, Frank Sparing

**Affiliations:** 1 Institut für Geschichte, Theorie und Ethik der Medizin, Centre for Health and Society, Heinrich-Heine-Universität Düsseldorf, Moorenstr. 5, 40225 Düsseldorf, Deutschland; 2 Oral-History-Forschungsstelle, Universität Erfurt, Steinplatz 2, 99085 Erfurt, Deutschland; 3 Institut für Neuere Geschichte, Heinrich-Heine-Universität Düsseldorf, Universitätsstraße 1, 40225 Düsseldorf, Deutschland; 4 Institut für Historische Bildungsforschung, Universität Hamburg, Von-Melle-Park 8, 20146 Hamburg, Deutschland

Wie in vielen anderen Disziplinen ist die Oral History eine weit verbreitete Methode der zeitgeschichtlichen Forschung im Allgemeinen und der medizinhistorischen Forschung im Besonderen. Neuere Interviewprojekte orientieren sich an aktuellen methodischen Standards und nutzen die verschiedenen Formen der medialen Darstellung. Bei genauerer Betrachtung wird jedoch deutlich, dass der methodische Zugang vor dem Hintergrund unterschiedlicher Wissensbestände und oft aus unterschiedlichen Fachkulturen heraus erfolgt.

Ausgehend von der These, dass sich Oral-History-Studien in der Medizingeschichte in einigen formalen, forschungsethischen und strukturellen inhaltlichen Aspekten von anderen biographiegestützten Projekten erheblich unterscheiden, diskutieren im vorliegenden Forum deutschsprachige Forschende der Oral History Herausforderungen und Potentiale der Oral History in der Medizin entlang folgender Fragen: Ist Oral History eine lohnenswerte Methode? Worin liegen die methodischen und praxeologischen Herausforderungen? Ist Oral History in der Medizin etwas Besonderes? Worin liegt der Mehrwert dieses historischen Zugangs?

Die folgenden Beiträge behandeln Aspekte wie Dilemmata zwischen forschungspraktischen Überlegungen (wie wissenschaftliche Transparenz und Nachvollziehbarkeit) und medizinethischen Konventionen (wie ärztliches Vertrauensverhältnis und Schweigepflicht) (Söhner et al.), biographiegestütztes Arbeiten mit vulnerablen Gruppen – Erfahrungsbericht (Sparing et al.), Rolle der Oral History und das Format der Narrativen Medizin (Arp) sowie Berührungspunkte in Projekten der Erziehungswissenschaft mit der Medizin (Schliwa).

## Forum: Oral History in Medicine. Something special?

As in many other disciplines, oral history is a widespread method of contemporary historical research in medical history and is of particular relevance to qualitative social research. More recent eyewitness projects are oriented towards current methodological standards and make use of the various forms of media representation. Nevertheless—as we will argue—oral history studies in medical history differ considerably in some formal, research-ethical and structural aspects of content.

Based on the thesis that oral history studies in the history of medicine differ considerably from other biography-based projects in some formal, research-ethical and structural aspects of content, in this forum German-speaking oral history researchers discuss the challenges and potentials of oral history in medicine with particular consideration of the following questions: Is oral history a worthwhile method? What are the methodological and praxeological challenges? Is oral history something special in medicine? What is the added value of this historical approach?

The following contributions deal with aspects such as dilemmas between practical research considerations (such as scientific transparency and traceability) and medical ethical conventions (such as medical confidentiality) (Söhner et al.), biography-based work with vulnerable groups—experience report (Sparing et al.), the role of oral history and the format of narrative medicine (Arp) as well as points of contact in educational science projects with medicine (Schliwa).

